# Antibacterial Activity of Peruvian *Tarasa* Species: A Comparative Study of the Antimicrobial Effects of Extracts From Three Different *Tarasa* Species, *T. capitata*, *T. operculata* and *T. tenuis*, Against Human Pathogens

**DOI:** 10.1155/ijm/5546594

**Published:** 2025-01-22

**Authors:** Carmen R. Yauri, Annette N. Trombert, Antonio M. Lazarte

**Affiliations:** ^1^Universidad Nacional de San Agustín, Arequipa 04000, Peru; ^2^Escuela de Biotecnología, Facultad de Ciencias, Ingeniería y Tecnología, Universidad Mayor, Santiago 8580745, Chile

**Keywords:** antimicrobial activity, *Escherichia coli*, GC/MS, methicillin-resistant *Staphylococcus aureus*, natural compounds, phytochemicals, *Staphylococcus aureus*, *Tarasa*

## Abstract

The growing problem of antibiotic resistance has driven the search for new sources of antimicrobial agents. Plants, particularly those from the Malvaceae family, have showed promising potential in this field. The present study is based on *Tarasa* extracts, and the antimicrobial action was assessed using *Staphylococcus aureus* and *Escherichia coli* as experimental bacterial strains. N-hexane extracts of *T. capitata, Tarasa operculata* and *Tarasa tenuis* were analysed and showed, for the first time, antimicrobial activities against human pathogens. GC/MS analysis identified several chemical compounds in the extracts that could be responsible for their antimicrobial activity. These findings suggest that *Tarasa* species could be a valuable source of new antimicrobial compounds.

## 1. Introduction

The misuse of antibiotics has resulted in the development of antibiotic-resistant bacteria, which poses a significant threat to human health and leads to increased treatment costs [1]. The rapid emergence of antibiotic-resistant bacteria is a global concern. According to the Centres for Disease Control and Prevention (CDC, https://www.cdc.gov), at least 1.27 million people worldwide have died due to the reduced effectiveness of these drugs. This resistance crisis is primarily attributed to the improper and excessive use of antibiotics, as well as a lack of new drug development within the pharmaceutical industry.

The CDC has classified several bacteria as urgent, serious, and concerning threats, putting patients at risk and imposing a growing clinical and financial burden on the healthcare system. Addressing this crisis requires coordinated efforts from different disciplines including research and the search for novel therapeutic substances [2].

Natural substances have evolved to interact with a wide range of target proteins, making natural sources an advantageous option for the search of new antimicrobial molecules. Pharmaceutical companies have always turned to natural materials as a starting point for drug discovery, as these materials have been capable of addressing many complex clinical issues. Coordinating efforts to conduct meaningful research and find new alternatives based on natural products is essential [3].

Natural products derived from essential oils and extracts are highly valued as they are extracted from various parts of plants, such as flowers, leaves, fruits, seeds, stems, roots, barks or resins. These are used as raw materials in various fields including perfumes, cosmetics, spices, food, nutrition and for the treatment of various health disorders [4].

The young leaves of Yuca or *Manihot esculenta* (Euphorbiaceae), Okra or *Abelmoschus esculentus* (Malvaceae), *Hibiscus acetosella* (Malvaceae) and *Pteridium aquilinum* (Dennstaedtiaceae) are traditionally used in folk medicine to alleviate fever and pain, as well as in the treatment of conjunctivitis, rheumatism, hemorrhoids and abscesses, demonstrating anti-inflammatory properties [5]. Similarly, *Sida* (Malvaceae) has been employed as a traditional remedy for diarrhoea, malaria, gastrointestinal dysentery, fevers, asthma and inflammation. Furthermore, n-hexane extracts of *Sida rhombifolia* exhibited relatively high pharmacological activities in anti-inflammation, cytotoxicity and anticholinesterase assays [6].

Plant extracts are important because they concentrate metabolites and secondary phytochemicals present in these plants, including alkaloids, polysaccharides, terpenoids, saponins and phenolic compounds (phenolic acids and flavonoids). These compounds possess antiallergic, antifungal, anti-inflammatory, antihypertensive, antiviral, antitumour, anticarcinogenic, antioxidant and antibacterial activities [7–13].

The Malvaceae family, consisting of approximately 243 genera and 4225 species, is widely distributed, primarily in tropical regions. Natural compounds derived from various Malvaceae species have gained global recognition for their therapeutic properties as their antioxidant, anti-inflammatory and antimicrobial activities [14]. In Perú, Malvaceae family is represented by 38 genera and 264 species, and endemic species can be found in various ecological regions, including the high-Andean, meso-Andean, and humid and dry Puna, at elevations ranging from 2500 m to 5100 m [15]. Some species had showed antimicrobial activity. For example, Abubidentin A, isolated from *Abutilon* species, had showed promising antibacterial activities against *Escherichia coli, Pseudomonas aeruginosa* and *Staphylococcus aureus* [16]. In the same way, acetone extracts from *Hibiscus sabdariffa* revealed bactericidal activity against multidrug pathogens as *Salmonella* and *E. coli* [17] and essential oil of *Pavonia* inhibit the growth of bacterial and fungi pathogens as *S. aureus, Diplococcus pneumoniae, E. coli, Klebsiella* sp*, Tricophyton mentagrophytes, Chrysosporium indicum, Aspergillus* sp, *Botryodiplodia* sp and *Fusarium solani* among others [18].

The Malvaceae genus *Tarasa* Phil. consists of 30 species that extend from central Perú to the central regions of Chile and Argentina. It has been classified into two sections: *Tarasa* and *Umbellata* [19]. Many species within this genus are used in traditional medicine but there are no data available regarding their potential as a source of antimicrobial substances. The objective of this study aims to compare, for the first time, the antimicrobial effects of extracts derived from three different species of the *Tarasa* genus, namely, *Tarasa capitata*, *Tarasa operculata* and *Tarasa tenuis*, against the human pathogens *S. aureus* ATCC25923, methicillin-resistant *S. aureus* (MRSA) and *E. coli*. The identification of chemical compounds with a higher antimicrobial effect was performed using gas chromatography/mass spectrometry (GC/MS).

## 2. Materials and Methods

### 2.1. Study Site

**Figure 1:**
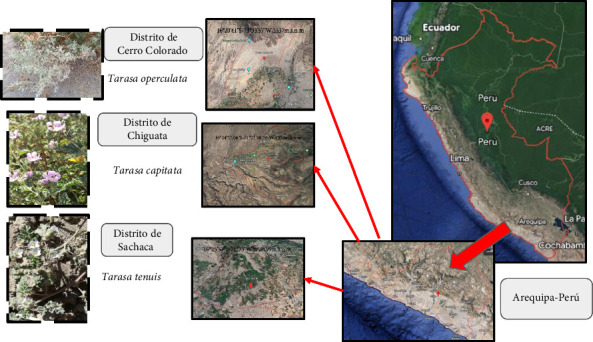
The map of Perú displays the study region, which is Arequipa, highlighting different sampling points such as Cerro Colorado, Chiguata and Sachaca. The figure illustrates the various *Tarasa* species found at each collection location.

*Tarasa* samples were collected from Arequipa, Perú, during November to December 2022. *T. operculata* was obtained from the Cerro Colorado district at coordinates 16°20′41″S–71°33′57″W, with an elevation of 2537 m above sea level (m.a.s.l.). *Tarasa capitata* was collected from the Chiguata district at coordinates 16°24′27.06″S–71°22′48.24″W, at an elevation of 3093 m.a.s.l. Lastly, *T. tenuis* was gathered from the Sachaca district at coordinates 16°25′53″S–71°35′36″W, situated at an elevation of 2199 m.a.s.l (Figure 1).

### 2.2. Preparation of *Tarasa* Extracts

Samples of *Tarasa* were collected in the morning hours in order to obtain a higher concentration of active principles [20]. Subsequently, the samples were washed with distilled water and dried in the shade for 2 weeks. Ten grams of each part of the plant under study (leaves, stems and flowers) were pulverised and macerated in 80 mL of n-hexane for 72 h [21].

Then, the samples were removed and placed on a filter paper for subsequent extraction in the Soxhlet apparatus (EUROLAB). The extracting solvent was heated in the flask, and its vapours were condensed on the filter paper, extracting the soluble analytes. This process was repeated until the extraction of the analytes from the sample was completed, and then they were concentrated in the solvent for 5 h. The dripping of the solvents was controlled to ensure the same number of extraction cycles for each of them. Finally, the *Tarasa* extract was obtained using a Soxhlet apparatus at 69°C.

The yield of the extract was calculated using the following formula [22].

(1) $$\begin{array}{c} \%R=\frac{Wi-Wf}{Wi}\times 100, \end{array}$$

where *%R* is the extract yield, *Wi* is the initial weight (g) and *Wf* is the final extract weight (g).

### 2.3. Bacterial Strains

*S. aureus* ATCC25923 and MRSA strains were obtained from Honorio Delgado Espinoza Hospital (Arequipa, Perú), isolated on mannitol salt agar (BBL). *E. coli* strain was obtained from a stool culture from a patient with diarrhoea and stomach pain. The identification of the strain was made using biochemical reaction–based identification, and the strain was subsequently cultured on MacConkey agar (BBL). Both strains were cultivated at 37°C at aerobic conditions for 24 h or until colonies were visible.

### 2.4. Disk Diffusion Test

The susceptibility of *S. aureus,* MRSA and *E. coli* to *Tarasa* extracts was assessed using the disk diffusion test method. Mueller–Hinton agar plates (MH, HIMEDIA) were prepared. Using a sterile inoculating loop, isolated colonies of each organism were touched, transferred and suspended in 2 mL of sterile saline solution (0.9% NaCl) to achieve a turbidity corresponding to a 0,5 McFarland standard. A sterile swab was immersed in each inoculum tube to obtain a bacterial suspension, which was then used to inoculate the dried surface of MH plates.

Sterile paper discs were positioned onto the agar surface using sterile forceps. Four different volumes of *Tarasa* extract—5 μL, 10 μL, 15 μL y and 20 μL—were impregnated onto separated discs. As control, equivalent volumes of n-hexane (EMSURE) and gentamicin 0.3% (GENTILE, Medifarma) were used. The inoculated plates were subsequently incubated aerobically at 37°C for 24 h.

Four repetitions of each assay were conducted. The data underwent analysis of variance (ANOVA), followed by Bonferroni post hoc test (⁣^∗^*p* < 0.05, ⁣^∗∗^*p* < 0.01 and ⁣^∗∗∗^*p* < 0.001) using Minitab statistical software (MINITAB 19) and RStudio (Posit) software.

### 2.5. Broth Macrodilution Test

To test if *Tarasa* extracts inhibit or kill pathogen bacteria, a broth macrodilution test was assessed. For this, the extract with the best antimicrobial effect was selected corresponding to *T. operculata* leaves, stems and flowers extracts. The procedure involved preparing 10 fold dilutions of extracts (1000 μg/mL, 100 μg/mL, 10 μg/mL, 1 μg/mL y and 0.1 μ/mL) in the Mueller–Hinton broth (HIMEDIA) dispensed in sterile tubes. Using a sterile inoculating loop, isolated colonies from *E. coli* and *S. aureus* agar cultures were touched, transferred and suspended in 2 mL of sterile saline solution (0.9% NaCl) to achieve a turbidity corresponding to a 0.5 McFarland standard. A volume of each standardised bacterial culture was used to inoculate each dilution and incubated at 37°C for 24 h. After the incubation period, turbidity was verified, each dilution was inoculated in Müller–Hinton agar plates and incubated at 37°C for 24 h.

### 2.6. GC/MS

The identification of chemical components of n-hexane extracts from leaves, flowers and stems of *T. operculata* was conducted by SLab Perú (https://slabperu.com/). The analysis was performed using SHIMADZU gas chromatograph, model GC-2010 Plus, autosampler SHIMADZU, model AOC-6000 and mass spectrometer SHIMADZU GCMS-QP2010 Ultra.

## 3. Results

### 3.1. Extraction Yield

In Table 1, the extraction yield percentage was obtained from each part of the studied *Tarasa* species. It is observed that the highest yield was generally obtained from the leaves, with an average value of 2.27%. Although a higher extraction yield may indicate a greater amount of extractable compounds in terms of weight, this does not necessarily imply greater antimicrobial activity. Extracts from leaves, stems and flowers may contain different profiles of bioactive compounds, which influence their antimicrobial efficacy.

This yield analysis serves as an initial reference for the extraction efficiency of each part of the plant. However, it is important to note that the specific composition of the compounds in each extract was subsequently analysed by GC to identify those responsible for the antimicrobial activity.

### 3.2. Disk Diffusion Test

In Table 2, the results of the disk diffusion test are shown. The diameters of the inhibition zones were obtained using a volume of 20 μL. Diameters obtained from lower volumes were smaller, and the results were not presented.

**Figure 2:**
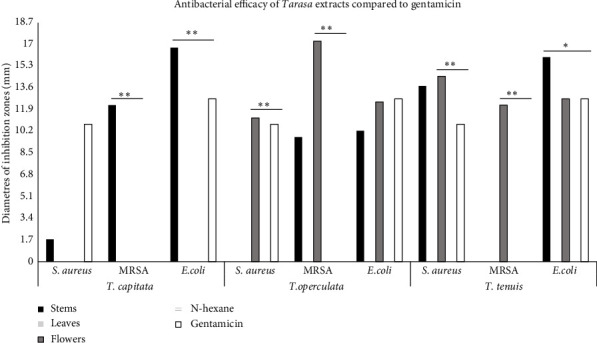
Inhibition zones comparing antibacterial efficacy of different *Tarasa* species against pathogen bacterial strains relative to gentamicin. ⁣^∗^*p* = 0.001 and ⁣^∗∗^*p* < 0.001.

Figure 2 shows that stem extracts from *T. capitata* were significantly more efficient than gentamicin (*p* < 0.001) in inhibiting *E. coli*. In contrast, flower extracts from *T. operculata* and *T. tenuis* showed significant efficacy against *S. aureu*s and MRSA. Specifically, the flower extract from *T. operculata* was notably more effective than gentamicin in inhibiting MRSA (*p* < 0.001). Notably, in the case of *E. coli*, stem extracts from *T. tenuis* exhibited superior inhibitory properties and outperformed gentamicin (*p* = 0.001).

### 3.3. Broth Macrodilution Test

The results demonstrated that *T. operculata* extracts killed *S. aureus* ATCC25923. Nevertheless, in *E. coli,* colonies were observed in dilution 1 μg/mL and 0.1 μg/mL, suggesting the MIC value of 1 μg/mL.

### 3.4. GC/MS

**Figure 3:**
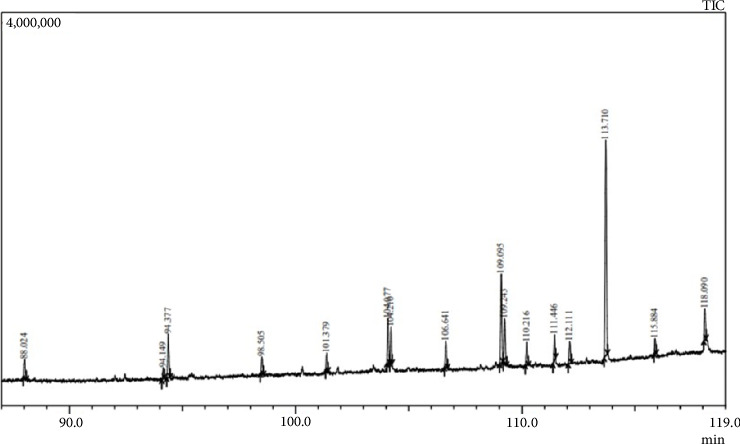
Gas chromatography/mass spectrometry (GC/MS) of *Tarasa operculata* extract.

In light of the observed efficacy of *T. operculata* in inhibiting bacterial strains, particularly MRSA, and the ability of its flower extracts to exhibit a pronounced antimicrobial effect against *S. aureus*, as well as the notable inhibitory activity against *E. coli* in both flower and stem extracts, it was decided to subject the extracts of this species to the GC/MS analysis. The results are shown in Figure 3.

The characterisation and identification of each peak from GC/MS is shown (Table 3).

The relative area of the compound in a GC/MS analysis (Area %) is directly proportional to the quantity of a specific compound present in the analysed extract. Thus, the results of the analysis of the *T. operculata* extract indicate that the predominantly detected compounds are linear alkanes such as tetracosane and pentacosane, with heneicosane being present in a lower percentage. Thus, several ethyl esters of fatty acids, saturated hydrocarbons and squalene, an organic compound found in high concentrations in certain vegetable oils, were identified.

## 4. Discussion

The yield of an extract is important as it indicates the quantity of active compounds obtained from a given amount of plant material. A higher yield results in a higher concentration of active compounds [23, 24]. However, there are studies suggesting that the relationship between yield and the number of active compounds in plant extracts is not always linear or direct [24, 25]. In the case of the results obtained in this study, it was observed that the highest yield was found in the leaves of *Tarasa*, but this does not correlate with high levels of the antimicrobial effect. Factors such as excessively high temperatures could lead to reduced activity, trigger the degradation of thermosensitive compounds and enhance the solubility of impurities [24]. Furthermore, the extraction time significantly impacts both yield and the concentration of active compounds within the extract. This effect may peak at a specific time before diminishing notably [23]. For *Tarasa* leaves, despite a 5-h extraction duration, it is possible that this was not the optimum period for extracting the active principles.

In the present study, the significant efficacy of flower extracts from *T. operculata* and *T. tenuis*, both belonging to the Malvaceae family, against *S. aureus* and MRSA was prominently highlighted. These findings corroborate prior evidence from Farasayu et al. [26], who also investigated flower extracts of *Hibiscus rosa-sinensis L*., another member of the Malvaceae family, and identified antibacterial properties capable of inhibiting the growth of *Streptococcus sanguinis*. Furthermore, Al-Saggaf [27] addressed the biosynthesis of extracts from calyces of *H. sabdariffa*, revealing their ability to combat multidrug-resistant pathogenic bacteria, including *Klebsiella pneumoniae*, *Salmonella typhimurium* and *S. aureus* through nanoconjugation between fungal nanochitosan and selenium nanoparticles. Periasamy et al. [28] also contributed to this line of research, demonstrating the antimicrobial activity of silver nanoparticles extracted from various parts of *Hibiscus rosa-sinensis*, including leaves, flowers and bark. Their results indicated higher effectiveness against *E. coli*, followed by *P. aeruginosa*, *S. aureus* and *Bacillus subtilis*.

In contrast, our results did not reveal any antimicrobial activity in *Tarasa* leaves for any of the three species studied. This observation is consistent with the conclusions of Rezaei [29], who assessed hydroalcoholic leaf extracts of *Althaea officinalis* L. (Malvaceae) and found them ineffective against Gram-negative bacteria but with positive effects against Gram-positive bacteria, including *S. aureus*. The above is reinforced by the broth macrodilution test where the extracts of *T. capitata* were able to eliminate the Gram-negative bacteria in each of the dilutions but not the Gram-positive.

It is important to note that, although our results showed a more pronounced antimicrobial effect against Gram-positive bacteria, such as MRSA, previous studies, such as the one conducted by Márquez et al. [30], have demonstrated promising results against Gram-negative bacteria when analysing the antimicrobial activity of *H. sabdariffa* L. (Malvaceae) extracts. Their research included in vitro tests to determine the minimum inhibitory concentration and minimum bactericidal concentration and obtained the best results, especially against Gram-negative bacteria.

Consistent with these investigations, our findings also suggest that stem extracts of *T. capitata* and *T. tenuis* may exert antimicrobial effects on Gram-negative bacteria. This broadens the spectrum of potential applications for these extracts in the treatment of infections caused by a variety of pathogenic microorganisms.

On another article, the work of Portillo et al. [17] should be mentioned, which focused on acetone extracts of calyces from *H. sabdariffa* (Malvaceae). Their study delved into the antimicrobial properties of these fractions and assessed their antimicrobial effect against multidrug-resistant strains of *Salmonella* and pathogenic *E. coli*. This research highlights the diversity of approaches within the Malvaceae family to combat bacterial infections and underscores the relevance of the ongoing search for therapeutic alternatives against the growing antimicrobial resistance.

Our results strengthen the evidence of the efficacy of Malvaceae flower extracts, as well as the importance of exploring different parts of these plants and their antimicrobial properties in the quest for new therapeutic strategies against bacterial infections. These findings provide a valuable contribution to the field of antimicrobial research and open new perspectives for future studies in this area.

In our study, several compounds were identified in the mixture of leaves, stems and flowers of *T. operculata*, including linolenic acid, squalene, hexadecanoic acid and saturated hydrocarbons. Previous studies by Ohta et al. [31] have demonstrated the antibacterial activity of *α*-linolenic acid against *S. aureus* in methanol extracts from the HS-101 strain of *Chlorococcum* and *Dunaliella primolecta* (MRSA). Furthermore, Kim et al. [32] found linolenic acid in tomato leaf extracts, which have also exhibited antimicrobial activity.

Regarding squalene, according to Dmitrieva et al. [33], this compound possesses antimicrobial properties. Squalene emulsion has showed high activity against *E. coli* B-8208, acting on biomolecules leading to oxidative stress. In addition, squalene nanoemulsions studied by Fang et al. [34] exhibit greater antimicrobial activity against Gram-positive bacteria than Gram-negative bacteria and fungi. It has been observed that the death of MRSA is primarily induced by direct damage to the cell membrane, leading to the leakage of proteins and cytoplasmic DNA. Nanoemulsions could also degrade DNA helix and disrupt protein synthesis.

Our results suggest that *T. operculata* extracts could be a potential source of antimicrobial compounds, including linolenic acid and squalene, which could be used as therapeutic agents against Gram-positive bacteria. However, further studies are needed to better understand the mechanisms behind the antimicrobial activity of these compounds and their potential use in combating antibiotic resistance.

Within the framework of this study, the impact of *Tarasa* extract on two fundamental categories of bacteria, namely, Gram-positive and Gram-negative, has been examined. These results significantly contribute to the fields of microbiology and phytochemistry by shedding light on the complex interactions underlying the relationship between natural compounds and microorganisms. The primary purpose of this research lies in the ongoing quest for effective approaches to combat bacterial infections. In accordance with previously documented observations by Knapp and Melly [35], it has been confirmed in this study that *Tarasa* extract exerts a more pronounced effect on Gram-positive bacteria in contrast to its response in Gram-negative bacteria. In addition, the findings of Nascimento et al. [36] in their comprehensive evaluation of the antimicrobial activity of plant extracts and phytochemicals reveal that these compounds are effective against both Gram-negative and Gram-positive bacteria. This contrast in results underscores the diversity and richness of natural compounds present in plants, emphasising the need for further investigation to fully understand variations in microbial response.

## 5. Conclusions

The antimicrobial effect of n-hexane extracts of flowers from *T. operculata* and *T. tenuis* was determined. They exhibited a significant antimicrobial effect against Gram-positive and Gram-negative strains, while the n-hexane extract of leaves showed no activity against any of the species tested. Antimicrobial activity was also observed in the n-hexane extract of stems in the three species, such as *T. capitata* (*S. aureus* ATCC25923, *S. aureus* MRSA and *E. coli*) and *T. operculata* and *T. tenuis* (*S. aureus* ATCC 25923 and *E. coli*).

Of the three plant species, *T. operculata* showed the greatest antimicrobial effect, and some of its chemical components were identified, such as linolenic acid, ethyl ester, squalene, hexadecanoic acid, ethyl ester and saturated hydrocarbons, which may be responsible for its powerful antimicrobial property. The results of this study suggest that extracts from *Tarasa* species could be used as natural sources of antimicrobial compounds. The importance of evaluating different parts of the plant to determine its antimicrobial potential and of identifying the chemical components responsible for its activity is highlighted. These findings may have important implications for the development of new natural antimicrobial agents and their application in the food and pharmaceutical industries.

## Figures and Tables

**Table 1:** Extraction yield of *Tarasa* species in different parts of the plant.

*Tarasa* species/yield (%)	Stems	Leaves	Flowers
*T. capitata*	1.21	2.07	1.47
*T. operculata*	1.12	2.76	1.64
*T. tenuis*	1.81	1.99	1.73

**Table 2:** Diametres of inhibition zones (mm) resulting from the exposure of bacterial strains to 20 μL of different extracts from *Tarasa* along with standard errors.

*Tarasa* species	Bacterial strains	Stems	Leaves	Flowers	N-hexane	Gentamicin
*T. capitata*	*S. aureus*	1.75 ± 0.5	0	0	0	10.75 ± 0.5
	MRSA	12.25 ± 1	0	0	0	0
	*E. coli*	16.75 ± 2	0	0	0	12.75 ± 0.5

*T. operculata*	*S. aureus*	0	0	11.25 ± 0.5	0	10.75 ± 0.5
	MRSA	9.75 ± 6.5	0	17.25 ± 2	0	0
	*E. coli*	10.25 ± 0.5	0	12.5 ± 1	0	12.75 ± 0.5

*T. tenuis*	*S. aureus*	13.75 ± 0.5	0	14.5 ± 0.5	0	10.75 ± 0.5
	MRSA	0	0	12.25 ± 0.5	0	0
	*E. coli*	16 ± 0.5	0	12.75 ± 0.5	0	12.75 ± 0.5

**Table 3:** Characterisation of GC/MS results.

Peak#	R. time	Area (%)	Height (%)	Name
1	88,024	3.17	2.79	Hexadecanoic acid, ethyl ester
2	94,149	1.5	1.69	Linoleic acid ethyl ester
3	94,377	6.82	6.43	9,12,15-Octadecatrienoic acid, ethyl ester, (Z,Z,Z)-
4	98,505	2.5	2.56	Heneicosane
5	101,379	2.35	2.61	Pentacosane
6	104,077	5.98	6.56	Pentacosane
7	104,21	4.81	5.28	Carbonic acid, 2-ethylhexyl octyl ester
8	106,641	3.81	3.91	Pentacosane
9	109,095	12.98	13.04	Pentacosane
10	109,243	6.6	6.52	Carbonic acid, 2-ethylhexyl undecyl ester
11	110,216	3.31	3.37	1,4-Benzenedicarboxylic acid, bis(2-ethylhexyl)ester
12	111,446	3.22	3.69	Tetracontane
13	112,111	3.37	3.25	Squalene
14	113,71	33.47	31.59	Tetracontane
15	115,884	1.98	2.17	Pentacosane
16	118,09	4.12	4.53	Tetracontane
		100	100	

## Data Availability

The data used to support the findings of this study are included within the article, and any additional data required are available from the corresponding author upon request.
